# Supplementary material for: Modern Technologies of Solution Nuclear Magnetic Resonance Spectroscopy for Three-dimensional Structure Determination of Proteins Open Avenues for Life Scientists
Source: Comput Struct Biotechnol J. 2017 Apr 13;15:328–39. doi: 10.1016/j.csbj.2017.04.001 (PMC5408130; doi:10.1016/j.csbj.2017.04.001)
Supplement: Supplementary file 1 — Supplementary material. [file mmc1.docx]

Modern Technologies of solution nuclear magnetic resonance spectroscopy for Three-Dimensional Structure Determination of Proteins Open Avenues for Life Scientists

Toshihiko Sugiki*, Naohiro Kobayashi, and Toshimichi Fujiwara

Institute for Protein Research, Osaka University

3-2 Yamadaoka, Suita, Osaka 565-0871, Japan

*Corresponding author:

Toshihiko Sugiki, Ph.D.

Specially Appointed Assistant Professor

Laboratory of Molecular Biophysics

Institute for Protein Research, Osaka University

3-2 Yamadaoka, Suita, Osaka 565-0871, Japan

TEL: +81-6-6879-4417

FAX: +81-6-6879-8599

e-mail: [sugiki@protein.osaka-u.ac.jp](mailto:sugiki@protein.osaka-u.ac.jp)

**Abstract**

Nuclear magnetic resonance (NMR) spectroscopy is a powerful technique for structural studies of chemical compounds and biomolecules such as DNA and proteins. Since the NMR signal sensitively reflects the chemical environment and the dynamics of a nuclear spin, NMR experiments provide a wealth of structural and dynamic information about the molecule of interest at atomic resolution. In general, structural biology studies using NMR spectroscopy still requires a reasonable understanding of the theory behind the technique and experience on how to recorded NMR data. Owing to the remarkable progress in the past decade, we can easily access suitable and popular analytical resources for NMR structure determination of proteins with high accuracy. Here, we describe the practical aspects, workflow and key points of modern NMR techniques used for solution structure determination of proteins. This review should aid NMR specialists aiming to develop new methods that accelerate the structure determination process, and open avenues for non-specialist and life scientists interested in using NMR spectroscopy to solve protein structures.

**Keywords**

Nuclear magnetic resonance (NMR) spectroscopy

Solution NMR

Automation

Protein

Structure determination

Validation

**Contents**

Introduction

1. Workflow of protein structure determination by solution nuclear magnetic resonance (NMR) spectroscopy

2. Protein sample preparation for NMR measurements

3. Acquisition of NMR spectra and their assignment

4. NMR structure calculation

4.1. Distance restraints

4.2. Dihedral angle restraints

5. Automated NOE assignment and structure modeling of proteins

6. Validation of precision and accuracy of the determined protein structure

7. Concluding remark

Acknowledgements

References

**Introduction**

Tertiary structure determination of biomolecules such as nucleic acids and proteins at atomic resolution provides essential insight into the function of bioactive molecules. X-ray crystallography [1] and nuclear magnetic resonance (NMR) spectroscopy [2] have been the primary methods over the past few decades to obtain high-resolution structures. More recently, the rapid technological growth of cryo-electron microscopy [3] has seen this technique emerge as a third major approach to solve biomacromolecular structures at atomic resolution.

Solution NMR offers a number of distinct features for structural biology studies: 1) Dynamics of protein folding, structural fluctuations, internal mobility and chemical exchange of target molecules can be investigated over a wide range of timescales (i.e., between picoseconds and submillisecond timescales) [4, 5].. The most successful application of NMR to study biomolecular dynamics has been carried out on intrinsically disordered proteins (IDPs) such as zinc finger proteins that usually do not yield crystals, which provide sufficient quality X-ray diffraction data for high-resolution structure determination [6-11]. Moreover, these proteins are too small or too flexible to obtain strong contrast images by modern cryo-EM analysis. 2) Studies of protein-protein or protein-ligand interactions can be performed under physiological conditions. The affinity and the location of the interaction sites between the target protein and its binding partner molecules can be determined accurately and sensitively even if the interaction is very weak (*K*_d_ = ~mM) by performing simple NMR experiments, e.g., chemical shift perturbation analysis by measuring two-dimensional (2D) heteronuclear single-quantum coherence (HSQC) spectra of target proteins with and without the binding partner, while such an interaction study by co-crystallization or co-immunoprecipitation can be challenging [12-14]. Therefore, techniques based on using solution NMR can be readily used to screen chemical fragment libraries for preliminary hits, which have the potential to act as seed compounds in drug development. Such approaches usually identify weak binders for the target protein, but represent a powerful starting point for structure-based drug discovery and development studies. Among the various applications of solution NMR to study protein function, as aforementioned, tertiary structure determination of proteins by NMR remains a steadfast and powerful application of this spectroscopy. Recent development of software and a number of web-based resources, which reduce the burden of complex NMR data analysis, have contributed to the systematic integration of sophisticated semi-automated NMR platforms for structure determination of biomolecules [15-19]. Such advances, along with improvements in NMR hardware, have lowered the knowledge barrier to facilitate entry into the field of structural biology by NMR for non-specialists. However, only a handful of articles that describes the workflow and practical aspects of protein structure determination by solution NMR spectroscopy, which covers the latest method developments, have been published despite the practicality of this technique.

**1. Workflow of protein structure determination by solution NMR**

The standard approach to protein structure determination by solution NMR comprises several steps: (i) preparation of a protein sample labeled with stable NMR spin-1/2 isotopes, ^13^C and ^15^N; (ii) acquisition of NMR spectra; (iii) data processing and assignments of signals; (iv) generation of a chemical shift table that gives the signal assignments derived from the analysis of NMR spectra such that the analyst or an automated program can subsequently assign NOE signals to generate distance or dihedral angle restraints; and (v) simulated annealing (SA) by simplified molecular dynamics calculation with the NMR-based restraints are used for initial structure modeling to obtain an ensemble of structures that satisfy the experimentally determined constraints. These SA calculations are followed by molecular dynamics (MD) simulation with explicit or implicit water using a more sophisticated force field. (vi) The final stage of the workflow is validation of the precision and accuracy of the determined structure (Fig. 1). Details on these steps are explained in the subsections below. In this mini-review, basic and general information for researchers interested in NMR structure determination of proteins will be provided. We will mainly describe general procedures that are routinely used for NMR structure determination of soluble and small-to-medium size (c.a. <25 kDa) proteins. More advanced techniques required for NMR analysis of more challenging proteins, e.g. membrane proteins or larger proteins (c.a. >25 kDa), will also be described briefly.

**2. Protein sample preparation for NMR measurements**

In the initial NMR studies aimed at solving protein structures, complete assignment of the proton (^1^H) NMR signals was achieved by acquiring ^1^H NMR spectra, e.g., 2D ^1^H–^1^H double quantum filtered-correlation spectroscopy (DQF-COSY), 2D ^1^H–^1^H totally correlation spectroscopy (TOCSY), and 2D ^1^H–^1^H nOe correlated spectroscopy (NOESY) spectra. These spectra provided sufficient information to obtain ^1^H–^1^H distance restraints to calculate the tertiary structure for non-isotope labeled peptides and small proteins (c.a. < 10 kDa) [20, 21]. Extension of the ^1^H only approach to larger proteins was not possible because of severe signal overlap precluding unambiguous assignment of signals [21]. In the early 1990s, owing to the development of technologies for expressing recombinant proteins using bacteria cultures with medium containing stable isotopes, a large number of target proteins could be uniformly labeled with the stable “NMR active” isotopes, ^13^C and ^15^N, which can be detected by NMR measurements. Two- (2D), three- (3D) and four- (4D) dimensional NMR spectra observing signals that correlated ^1^H, ^13^C and ^15^N nuclei made for dramatic improvements in signal separation and removed signal overlap present in ^1^H NMR spectra [21].

The *Escherichia coli* (*E. coli*) expression system is most commonly used for stable isotope labeling of proteins, as it has several advantages: easy handling, bacterial cell grow quickly, cost efficiency and the availability of many established methods for protein isotope labeling [22, 23]. For example, the most powerful application of the methods would be site-specific/desired amino acid selective isotope labeling of proteins, which has been firmly established using the *E. coli* expression system [22-25]. Alternative ^13^C-labeling of protein is also possible by using particular ^13^C-enriched carbon sources, such as [1-^13^C]-glucose [26], , [1,3-^13^C_2_]- or [2-^13^C]-glycerol [27], ^13^C-acetate [28], [1,2-^13^C_2_]- or [3-^13^C]-pyruvate [29-31]. As this technique enables NMR observation of only the desired moiety of the target protein, it is especially useful for large molecular-weight (c.a. > 25 kDa) proteins or IDPs that show severe signal degeneracy.

In situations were insufficient amount of the recombinant proteins is produced by the *E. coli* expression system, the yeast expression systems, *Pichia pastoris* [32] or *Kluyveromyces lactis* [33] as host cells, can be used as an alternative. These systems not only have the advantage of being similar to the *E. coli* expression system (easy handling, rapid cell growth, and many available and firmly established isotope labeling techniques with relatively low cost), but also offer eukaryotic cell-specific features that facilitate the over-expression and correct folding of particular proteins, e.g., proteins requires a complex array of disulfide bonds to form for native folding [22, 23, 34, 35].

Cell-free protein expression systems are another option to overexpress proteins. This approach synthesize recombinant proteins *in vitro* in a reaction mixture containing the DNA cording the target protein and a cell lysate containing gene transcription/translation molecular machineries, and, if required, isotope labeled amino acids for protein labeling [36-40]. Preparation of a fresh cell lysate, which is necessary for synthesizing a sufficient amount of the recombinant protein stably and reproducibly, is challenging. Furthermore, the running cost of such a cell-free system is generally high [23], indicating that it is not suitable for large-scale protein production with stable isotopes. However, this approach is still adequate for amino acid selective isotope labeling or selective introduction of unnatural amino acid residues into the target protein, because amino acid scrambling is relatively mild when compared with other expression systems that use host cells [41-46]. As an additional remarkable point, reconstructing an isotope-labeled membrane protein into artificial lipid bilayer discs, e.g., nanodiscs, is relatively easy to achieve by cell-free expression [47-50].

Isotope labeling of recombinant protein using insect or mammalian cells is also possible, although this is generally an expensive method with low yield. Moreover, the available isotope labeling variation is limited when compared with that of other expression systems [51, 52].

In NMR protein structure determination, sample purity should be as high as possible because signal linewidth and protein stability are affected by non-specific contact with impurities. However, NMR spectra can be obtained even if the sample is crude, as demonstrated by studies examining cell lysate or metabolites [53-58].

The conditions of the protein solution and the NMR experimental parameters should be sufficiently optimized prior to starting multidimensional heteronuclear NMR measurements [59-61]. In many cases, the protein concentration, hydrogen ion (pH) value of the sample solution, the type of salt/buffer and its concentration and the NMR measurement temperature should be carefully examined by several pilot NMR experiments (typically 1D ^1^H or 2D ^1^H–^15^N HSQC spectra are recorded) with different sample solution compositions and NMR parameters. If an optimal condition is found, the NMR spectra will show sharp (narrow linewidths) and sufficiently well-dispersed signals [59, 61, 62]. To assign backbone ^1^H, ^13^C and ^15^N signals successfully by the inter-residue chemical shift linking approach, the observation of ^13^C^β^ nuclei with reasonable chemical shift dispersion and sensitivity is a key point to consider. Pilot measurements of 3D HNCACB and/or 2D HN(CA)CB spectra can be good indicators for judging the adequacy of the solution conditions and potential difficulties of obtaining signal assignments Perdeuteration of target proteins combined with transverse relaxation optimized spectroscopy (TROSY)-type NMR pulse schemes may be beneficial when the molecular weight of the target protein exceeds 25 kDa (see Section 3).

Although the sensitivity of solution NMR is continuously improving thanks to the development of new NMR pulse schemes and hardware, e.g., an increase in the static magnetic field strength and the innovation of cryogenic probe [63], a higher protein concentration is preferable to obtain NMR signals with higher signal-to-noise ratio and resolution within a reasonable period of NMR instrument use. In particular, for protein structure determination, sufficiently high solubility and stability are typically required. Ideally, the target protein should be a mono-dispersed species at a protein concentration >0.5 mM and be stable in the NMR instrument for a minimum of 1-5 days.

The aggregation character of the target protein can be monitored by observing broadening of the NMR signals as a function of protein concentration or by investigating the rotational correlation time of the target protein via measurement of the [^15^N,^1^H]-TRACT [64] or the ^15^N T_1_/T_2_ value [65]. Biochemical wet experiments (e.g., size-exclusion column chromatography) are also useful for investigating the character of the target protein. However, this method cannot address protein aggregation at the protein concentration used for NMR measurements, because the target protein is significantly diluted in chromatography [59].

In general, the pH value of the protein solution is selected by considering the theoretically/experimentally determined isoelectric point (pI) of the protein. A lower pH is preferable to reduce the chemical exchange between water ^1^H and protein ^1^H^N^ nuclei, because ^1^H fast exchange reduces the sensitivity of ^1^H^N^–^15^N correlation signals [66, 67]. In some cases, however, the protein cannot be placed at a low pH because of the pI, solubility, or stability. As buffer components, phosphate buffer (e.g. sodium or potassium phosphate) is an ideal choice because it does not have ^1^H nuclei [61]. As a character of buffer conductivity and its effect on the sensitivity of NMR signal detection using cryogenic probes, however, other buffers such as HEPES- or MES-NaOH would be more preferable [68].

The sample temperature during NMR data collection is often room temperature (typically 25°C). However, a higher sample temperature should give better spectra unless such temperatures affect the structure and stability of the target protein (up to 40°C when a cryogenic probe is used), Higher temperatures offer sharper signals by decreasing solvent viscosity and thus the rotational correlation time of the protein [59, 61].

Some types of chemical additives can increase the solubility and/or stability of proteins [59, 61]. For example, a reducing agent (e.g., dithiothreitol (DTT), tris(2-carboxyethyl)phosphine(TCEP)-HCl) may be essential for preventing protein multimerization caused by inter-molecular nonspecific disulfide bond formation, when free cysteines exist on the surface of the protein. Multiple well plate-based high-throughput screening methods using fluorescence correlation spectroscopy or thermal shift assays can explore chemical additives that improve protein solubility and stability, which may help to find the best condition without exhaustive sample condition optimization work that consumes limited NMR instrument availability[59, 61].

Note that 5%–10% (v/v) ^2^H_2_O must be added into the NMR sample to provide a lock signal for the static magnetic field passing through the sample solution. In addition, in many cases, a standard chemical shift reference compound is dissolved in the sample solution, as described in the next section.

**3. NMR signal acquisition and their assignments**

Initially, ^1^H, ^13^C, ^15^N NMR signals arising from the nuclei of the target protein have to be assigned before we can obtain angle and distance restraints for protein solution structure determination. In general, a number of NMR spectra are recorded to obtain a complete set of NMR assignments [69-71].

For backbone (^1^H, ^13^C and ^15^N) signal assignments: 2D ^1^H–^15^N HSQC, 3D HNCA*, 3D HN(CO)CA*, 3D HNCACB, 3D CBCA(CO)NH, 3D HNCO, 3D HN(CA)CO*, and 3D HBHA(CO)NH are recorded. The 3D HNCACB and 3D CBCA(CO)NH are the most basic spectra for backbone signal assignments [71]. The asterisk next to the experiment acronym indicates optional spectra required when 3D HNCACB and 3D CBCA(CO)NH spectra are insufficient for completing backbone assignment. The 3D HNCO and 3D HN(CA)CO are useful complements to inter-residue chemical shift linking using 3D HNCACB and 3D CBCA(CO)NH experiments, and also help to eliminate any potential assignment unambiguity that exists. Furthermore, chemical shift assignments of ^13^C^α^, ^13^C^β^, ^13^C' and ^1^H^α^ nuclei is important for secondary structure prediction by TALOS or its related programs (e.g., TALOS+, TALOS-N) as described in Section 4.2. In those pulse schemes, except CBCA(CO)NH, ^1^H–^13^C–^15^N correlations are measured using the out-and-back coherence transfer approach between ^1^H and ^13^C/^15^N to improve the sensitivity of those low gyromagnetic ratio (γ) nuclei (typically 5–10 fold). First, polarization of proton magnetization is transferred to low-sensitive nuclei ^13^C or ^15^N via *J*-coupling, and then the chemical shifts of those nuclei are recorded. Finally, the magnetization of ^13^C or ^15^N are returned to the starting proton, and the chemical shifts of the ^1^H nuclei are recorded as the free induction decay (FID). However, nuclei denoted within brackets in the acronym of the pulse sequence participate in the coherence transfer pathway but their chemical shifts are not encoded in those multi-dimensional NMR spectra. Incidentally, those out-and-back coherence transfer schemes starting from the amide proton are also applicable to perdeuterated proteins.

For side chain assignment: 2D ^1^H–^13^C constant-time (CT) HSQC (^13^C offset on aliphatic and aromatic regions), 3D ^15^N-edited TOCSY-HSQC, 3D CC(CO)NH, 3D H(CCCO)NH, 3D (H)CCH-TOCSY (^13^C offset on aliphatic and aromatic regions), 3D H(C)CH-TOCSY (^13^C offset on aliphatic and aromatic regions), 2D (HB)CB(CGCD)HD, 2D (HB)CB(CGCDCE)HE, ^13^C-edited NOESY-HSQC and ^15^N-edited NOESY-HSQC experiments are required. There are many variations of those pulse schemes for time evolution such as CT or semi-CT methods, echo and anti-echo, single or multiple quantum coherence, or TROSY type acquisition. It is preferable that each user selects proper pulse schemes for individual protein samples by performing pilot NMR experiments to collect better quality NMR spectra that have the highest signal-to-noise ratio and resolution. Catalogues of standard pulse programs disclosed by the manufacturer of NMR instruments, such as Bruker (available from their websites), may help determine the best NMR measurements to use. The minimum number of multi-dimensional NMR spectra required to obtain complete assignment of ^1^H, ^13^C, ^15^N signals of proteins using magnetization transfer via inter-nuclear *J*-couplings is six: 3D HNCACB, 3D CBCA(CO)NH, 3D (H)CCH-TOCSY (aliphatic and aromatic regions) and 3D H(C)CH-TOCSY (aliphatic and aromatic regions) [71]. In practice, however, using NOESY spectra (e.g., ^13^C-edited NOESY-HSQC), which provide a wide range of inter-nuclear distance information between the backbone and side chains, may be helpful, in particular, for assignment of aromatic side chain signals.

The ^13^C- and ^15^N-edited NOESY-HSQC spectra are essential for both signal assignments and for generation of ^1^H–^1^H distance restraints [21, 72]. The mixing time used in NOESY spectra of proteins is generally 80–150 ms, as described in Section 4.1.

In general, when the molecular weight of the target protein exceeds 30 kDa, measurement and assignment of the protein NMR signals become difficult owing to the increasing degeneration and line-broadening of the signals, of which effect is associated with fast transverse magnetization decay [21]. The latter issue is the biggest burden to solution NMR spectroscopy. For example, the transverse relaxation rate of ^13^Cα magnetization is dominated by the dipole-dipole (DD) interaction with the proximate ^1^Hα, leading to dramatic sensitivity loss to ^13^Cα signals or signals in multidimensional NMR spectra that transfer magnetization via ^13^Cα. This is particularly problematic when the molecular weight of the target protein exceeds 20 kDa [73, 74]. In this case, the DD relaxation of ^13^Cα magnetization can be suppressed remarkably by substitution of ^1^Hα to ^2^H (perdeuteration) by overexpressing the target protein in ^2^H_2_O media [73, 74]. In addition, ^1^H-^15^N correlation spectra with narrower signals can be obtained by TROSY-type NMR pulse schemes, which selectively observes the coherence where DD relaxation has been attenuated by the chemical shift anisotropy (CSA) effect [75]. The TROSY effect will be more prominent in combination with a perdeuterated protein and higher static magnetic fields (i.e., ≥ 800 MHz) because cancellation of DD relaxation by the CSA effect is field dependent. However, the benefits from perdeuterated proteins are limited to improving backbone NMR signal assignments and for ^13^C-direct detection NMR experiments[76-69]. ^13^C-direct detection NMR methods are also applicable to protein structure determination [80]. In many cases, moreover, mild denaturation and regeneration treatments of the deuterated protein sample in a ^1^H_2_O buffer may be required to back-exchange deuterium to proton at amide positions to regenerate ^1^H^N^-^15^N correlations. However, multi-dimensional NMR experiments that use polarization transfer starting from non-labile protons, e.g. 3D CBCA(CO)NH, cannot be used with deuterated proteins [71].

Prior to starting the assignment of ^1^H, ^13^C and ^15^N chemical shifts of the target protein, the NMR spectra should be calibrated [81, 82]. ^1^H chemical shift calibration (or denoted as “referencing”) uses the temperature-dependency of the ^1^H chemical shift of ^1^H_2_O automatically when processing NMR spectra using the program NMRPipe [83]. In addition, chemical shift calibration using standard compounds that yield ^1^H chemical shifts, e.g., 4,4-dimethyl-4-silapentane-1-sulfonic acid-*d*6 (*d*6-DSS), offer further reliable calibration [81, 82]. Practically, calibration can be achieved by simply measuring the chemical shift value of the major ^1^H peak of the trimethyl group of *d*6-DSS (expected to appear near 0 ppm) in a 1D ^1^H spectrum. It is important for appropriate calibration to acquire the 1D ^1^H spectrum with sufficient number of data points, in order to read its peak maximum as precisely as possible. Then, the ^1^H carrier offset of the NMR spectra of the target protein is adjusted as the chemical shift value of the ^1^H peak of the trimethyl group of *d*6-DSS becomes 0.000 ppm. As shown in the BioMagResBank (BMRB) website (<http://www.bmrb.wisc.edu/ref_info/cshift.html>), the calibrated offset of the ^13^C and ^15^N carrier frequencies (Hz) of the target protein are determined by multiplying factors based on the gyromagnetic ratio (γ_13C_/γ_1H_ or γ_15N_/γ_1H_, those values correspond to 0.251449530 or 0.101329118, respectively) to the calibrated ^1^H center frequency (Hz) [81, 82]. After chemical shift referencing of the ^1^H, ^13^C, ^15^N center frequencies, all of the NMR spectra of target protein are processed.

It is also possible to obtain the 1D ^1^H NMR spectrum of a standard compound by dissolving it in a target protein sample solution as an internal standard. However, the user has to be careful that occasionally nonspecific interactions between the standard compound and the protein can occur. Furthermore, a strong ^1^H signal and ^1^H noise derived from the standard compound often obstructs analysis of the NMR spectra. Therefore, it is also acceptable to measure the ^1^H reference spectrum of the standard compound using a NMR sample only containing standard compound, e.g., 0.5mM DSS dissolved in 90% (v/v) H_2_O/10% (v/v) ^2^H_2_O, as an external standard.

Chemical shift referencing is mandatory for depositing the NMR data and structure into public databases such as the BMRB and Protein Data Bank (PDB). In addition, chemical shift calibration is important for accurate prediction of secondary structures and backbone dihedral angles by comparing data to chemical shift/structure databases using TALOS or related programs [84, 85], as described in Section 4.2. In any case, it is crucial to obtain reference chemical shift data on a standard compound at an identical temperature and static magnetic field strength to the NMR data collection on the target protein.

The processing of the collected NMR data involves a series of several mathematical conversions from time domain data to frequency domain data. Here, fast Fourier transformation of discretely sampled digital data of the direct and indirect dimensions with adequate shaping by apodization and window functions, linear prediction, phase adjustment and baseline correction are comprehensively achieved by programs such as NMRPipe (<https://spin.niddk.nih.gov/bax/software/NMRPipe/>) [83].

The chemical shift of the signals arising from the target protein should be assigned as completely as possible. In particular, ^1^H assignments are important, as they form the basis of inter-proton distance restraints. The completeness and accuracy of the ^1^H assignments will strongly and directly affect the accuracy and convergence of the calculated NMR structure [86-90]. There are many visualization software packages to examine and analyze the NMR spectra and perform chemical shift assignments, e.g., Sparky (Goddard TD and Kneller DG, SPARKY 3, University of California, San Francisco), CARA (developed by Dr. Keller of the Kurt Wüthrich’s group, <http://cara.nmr-software.org/portal/>), NMRView [91], Kujira and MagRO (developed by Prof. Naohiro Kobayashi of the PDBj-BMRB group (Osaka University, Japan), <http://bmrbdep.pdbj.org/en/nmr_tool_box/magro_nmrview.html>) [15, 17] with NMRViw and CcpNmr (<http://www.ccpn.ac.uk/>). Programs for automated assignment of backbone and side chain signals are also available, such as PINE and PINE-SPARKY, which is included in the new version of Sparky (NMRFAM-Sparky) [92, 93], MARS [94], UNIO (assembly of MATCH/ASCAN/CANDID/ATNOS algorithms) [87, 88, 95-97] and FLYA [98], which is partially powered by the GARANT algorithm [99]. The basic concept of the automated signal assignment process is matching peaks between experimentally observed and expected ones, which are based on the amino acid sequence of the target protein and magnetization transfer schemes of the recorded NMR spectra [99]. From a practical perspective, successful performance of automated assignment software requires the collection of high-quality NMR spectra that show sufficiently well-resolved signals and the expected number of signals. They typically provide reliable assignment of well-separated signals by linking chemical shifts, which are expected from coherence transfer pathways based on the pulse schemes used for the NMR experiments and topology of chemical structures of amino acid residues. Moreover, auto-assignment programs can offer possible assignment of unassigned signals, which are difficult to assign manually owing to ambiguity of inter-residue chemical shift linking. In many cases, therefore, the user has to manually confirm and carefully correct the assignment made by automated programs using NMR spectrum visualization software, as described above.

**4. NMR structure calculation**

**4.1. Distance restraints**

Protein structure determination by simulated annealing uses the ^1^H–^1^H distance and dihedral angle restraints derived from NMR data [86, 90]. The ^1^H–^1^H distance information is obtained by estimating the intensity of the ^1^H–^1^H NOE signals [21]: the intensity of the ^1^H–^1^H NOE signals are dependent on the mixing time (τ_m_), during which ^1^H–^1^H cross-peaks are generated [21]. However, although build-up rates obtained from linear dependency of the NOE cross-peak intensity on the τ_m_ correlate with the ^1^H–^1^H distance when a limited (short τ_m_) NOE build-up regime is used, quantitative estimation of ^1^H–^1^H distances from the intensities of the ^1^H–^1^H NOE signals becomes impossible over the limited τ_m_ range because relaxation and spin diffusion also proceed during long mixing times [21, 100]. In general, the τ_m_ value should be within the initial NOE build-up range (typically 80–150 ms for proteins) [21]. Nonetheless, recently developed innovative methods offering more exact conversion of NOE data to internuclear distance information, Exact NOE (eNOEs), are powerful protocols for accurate and precise protein solution structure determination [101-103]. The 3D ^13^C- or ^15^N-edited NOESY-HSQC spectra are widely used. Following peak picking and integration of the ^1^H–^1^H NOE signals, the table containing the residue number/atom name/chemical shift/^1^H–^1^H NOE signal intensity is used as primary input data for simulated annealing [90]. In the classical way to prepare NOE based distance restraints, the NOE peaks are roughly classified into three (or four) groups depending on their signal intensity: strong, medium and weak (and very weak) [86, 90]. In the structure calculation, this information is translated into ^1^H–^1^H distance ranges of 1.8–2.7, 1.8–3.3, and 1.8–5.0 (and 1.8–6.0) Å [86, 90]. The lower limit of the ^1^H–^1^H distance, 1.8 Å, corresponds to twice the van der Waals radius of ^1^H atoms [86, 90].

Rough classification and wide range (not rigorous) distance restrictions based on NOE data are based to the difficulty of converting the NOE signal intensity to an inter-nuclei distance accurately and directly because the NOE signal intensity is affected by not only the inter-atomic distance, but also various factors such as spin-diffusion and conformational averaging as a result of local structural fluctuations. Because of the mildness of distance restraints based on NOE experiments, a sufficient number of distance restraints are required for accurate and precise protein structure determination (generally speaking more than 10–20 distance restraints per residue, not including intra-residue NOEs) in order to build a sufficient density of NOE-network anchoring [90]. The concept of NOE-network anchoring is based on the consideration that the correctly assigned NOE distance restraints will form a self-consistent NOE set, which is compatible with tertiary structure models of target proteins [87-88]. It is also important to empower automated NOE assignment by the program CYANA [90] (see Section 5).

In particular, a sufficient number of long-range distance information (5.0–6.0 Å, which are upper limit distance restraints) is critical for precise and accurate structure determination [104]. As the intensity of NOE peaks with important distance information, i.e., long-range, is often weak, optimization of experimental condition of the NOESY spectra is important to gather a sufficient number of weak NOE signals. Higher sample concentration (>0.5 mM), lower temperature, lower pH (in the case of ^15^N-edited NOESY-HSQC) and stronger static magnetic fields are preferable parameters to ensure observation of weak NOEs. When only a minimal number of NOE signals are observed owing to the poor character of the target protein, e.g. aggregation or signal line broadening owing to averaging of multiple conformation caused by fluctuation in an intermediate-slow NMR time scale regime, long-range distance information can be collected, which may complement the shortage of NOE-based distance restraints, by protein labeling with paramagnetic metal ions or radicals to measure the paramagnetic relaxation enhancement (PRE) effect [105] or the pseudo-contact shift (PCS) [106, 107]. The PCS data provide important NMR-based restraints that describe the global fold of the protein and/or tertiary structure of protein-protein complexes with high accuracy owing to its unique features: PCS provides both long-range distance restraints and angle information of bond vectors in the protein against the tensor frame of the magnetically aligned paramagnetic center molecule.

In addition, the resolution of NOESY spectra significantly affects the quality of the structure determined by NMR [108]. Using the highest possible resolution is appropriate for precise and accurate structure determination; this is especially important when performing automated NOE peak picking and assignments. Algorithms for automated NOE peak picking, e.g., NMRView [91], AUTOPSY [109], ATNOS [88], AUDANA [110], or CYPICK [111], have been used for preparing peak lists to perform automated NMR analysis.

Information about the location of hydrogen bonds can be used as distance restraints [90]. The rate of chemical exchange between the amide proton in the protein and protons in the solvent water can be estimated by performing H/D exchange [85] or CLEANEX-PM [86] experiments. It is possible that amide protons showing a significantly slow exchange rate may be involved in the formation of hydrogen bonds. At a stage of the structure calculation process that the global fold of the protein has been trustfully determined, pair residues forming hydrogen bonds may be deduced from the modeled structure. However, this step must be carefully executed to prevent incorrect assignment of hydrogen bonded residues. More ideally, therefore, pair residues forming hydrogen bonds should be experimentally determined by long-range HNCO measurement [114, 115] or other analogous NMR techniques to avoid interpretation of the modeled structure. The distances between backbone amide protons and carbonyl oxygen atoms, or backbone nitrogen and carbonyl oxygen, are normally set as 1.8–2.0 or 2.7–3.0 Å, respectively. Since the distance restraints of hydrogen bonding are strong, giving large entropic information that contrasts those of dihedral angles and NOE-based distance restraints, introduction of hydrogen bonds should be applied at a later stage in the structure calculation. In a similar manner, information of the location of intra-molecular disulfide bonds also available as restraints should be entered carefully into a structure calculation, perhaps after an initial model has been calculated using purely distance and dihedral restraints.

**4.2. Dihedral angle restraints**

Dihedral angles of the target protein, such as backbone φ(N-C^α^), ψ(C^α^-C’), ω(N^α^-C’) and side-chain χ(n), are determined by a wide variety of NMR experiments [86, 90]. Those angles are important for defining the secondary structure and the side-chain conformation of the target protein.

According to the Karplus equation, a dihedral angle can be estimated by measuring the ^3^*J*-coupling constant [116]. For example, the ^3^*J*-coupling constants of each residue can be directly measured by NMR experiments such as 3D HNHA [117], ARTSY-*J* [118] and 3D HNHB [119]. However, acquiring these experiments with high precision and accuracy cannot be achieved easily because the sensitivity of those NMR experiments that measure ^3^*J*-couplings is relatively low. In those pulse schemes, long evolution and refocusing periods (total ~50 ms in the case of 3D HNHA) are required to measure the rather small ^3^*J*-coupling constants (< 10 Hz), which would be especially difficult to measure accurately with a protein that gives rise to broad signals owing to, for example, molecular size or chemical exchange processes [118]. As an alternative approach, dihedral angles and the secondary structure of proteins are predicted by the programs TALOS (and related versions) [84, 85], CamShift [120] and SHIFTX2 [121]. These programs use prior chemical shifts statistics, including ^1^H^N^, ^15^N, ^13^C^α^, ^13^C^β^, ^1^H^α^, and carbonyl ^13^C, with corresponding structure coordinates.

Dihedral angle information is useful to refine secondary structures in spite of their application in simulated annealing as relatively mild restraints (lower and upper limits are normally around +/– 20–40°).For application of the predicted dihedral angles in structure modeling, it is important to use trusted chemical shifts derived from correctly calibrated NMR spectra, as described above.

**5. Automated NOE assignment and Structure Modeling of Proteins**

Programs for automated NOE assignments and molecular modeling, e.g., CYANA [86, 90, 122], Xplor-NIH [123], AUREMOL (http://www.auremol.de/), critical assessment of automated structure determination of proteins by NMR (CASD-NMR) developed by the e-NMR project (<http://haddock.chem.uu.nl/enmr/eNMR-portal.html>) [19, 124, 125], PONDEROSA-C/S (http://ponderosa.nmrfam.wisc.edu/) developed by the National Magnetic Resonance Facility at Madison (NMRFAM) (<http://www.nmrfam.wisc.edu/software.htm>) [16, 18, 126] and UNIO (<http://perso.ens-lyon.fr/torsten.herrmann/Herrmann/Software.html>) [19, 87, 88, 95-97], are used for solution structure determination of biomolecules such as proteins and nucleic acids. In this mini-review, we will show an example of the program CYANA, which is used widely for NMR structure determination, to explain the general workflow of protein structure calculation. The amino acid sequence data of the target protein, the chemical shift table, and the NOE peak list are required as primary input files to perform automated NMR assignments and molecular modeling using CYANA [86, 92, 122]. In NOE based structure modeling by CYANA, seven steps of iterative calculations including the structure-modeling step for obtaining an initial global fold of the protein are performed. This is followed by refinements of the automated NOE assignments by referencing the calculated intermediate structure model of the protein using the algorithm extended from the program CANDID [90, 122]. The automated NOE assignment process has the advantages of being fast, unambiguous and unbiased [88, 127]. However, in many cases, manual intervention and/or re-assignment of the results of automated NOE assignment are necessary because completeness of the automated process is not perfect, especially when the peak maxima of the NOESY signals do not match ^1^H chemical shift assignment table [88, 127]. Several other algorithms, e.g., NOAH [128, 129], ASDP [130], ARIA and its related programs [131, 132], KNOWNOE [133], AutoNOE-ROSETTA [134] and PASD in Xplor-NIH [135], have also been developed for automated NOE assignments and structure calculation. Even if the stereo-specific assignments of methylene protons or methyl groups of leucine or valine residues are ambiguous, in the process of the structure-aided NOE auto-assignment, those signals can be automatically swapped with fair correctness by accounting for the modeled structure just before the final stage in the calculation. A high precision of modeled structures can be strongly supported by random combination of long-range distance restraints and sufficiently condensed NOE-network anchoring generated in the early stage of the CYANA calculation [87, 90].

In particular, when performing structure-aided NOE auto-assignment and structure modeling, the quality of the resultant structures strongly depends on the completeness and correctness of the assigned chemical shits [86-89]. Notably, a number of spurious NOEs such as noise peaks included in the input can lead to wrong NOE assignments, and thereby result in incorrect modeled structures. These issues have been intensively discussed in a literature [90]; nevertheless, a sufficient number of correct but weak NOE signals providing long-range distance information is a key feature for accurate and precise structure modeling [90]. In contrast, using the conventional approach of manual NOE assignments and generation of distance restraints, violations in the structure calculation caused by restraint violations may act as indicators to determine whether there are problems in the restraints list. Thus, in the conventional way, when systematic violations arise from the presence of incorrect restraints, the structure calculation and revision of restraints have to be repeated exhaustively. Both conventional and automated approaches can lead to endless and repetitive rounds of calculations, and thus a set of criteria is needed to ensure such a cycle is avoided. Previously, the national project Protein 3000 mainly performed by RIKEN solved a large number of structures by solution NMR using the CYANA and Kujira systems (over 1,080 structures) [15, 17]. This project revealed a number of benchmarks to achieve when solving structures by NMR: 1) the remaining number of unassigned NOE peaks should be < ~5%, which should not be localized to a certain region of the calculated structure; 2) high completeness and accuracy of the assigned chemical shifts of ^1^H, ^13^C and ^15^N signals (>90% of completeness); and 3) a sufficient number of NOE signals in the NOESY spectra (>10–20 NOEs per residue), with a wide intensity range and sufficiently high signal-to-noise ratio and resolution. Using the Kujira and MagRO system, the analysts can easily and visually inspect the accuracy of the assigned chemical shifts and the NOE signal that were automatically assigned by CYANA. In the process, NOE signals showing abnormal line-shapes and/or spurious NOE peaks can be eliminated. The aforementioned strategy should be suitable for structural studies of small proteins that give good-quality NMR spectra, which has been quantitatively evaluated by Kobayashi et al. [15, 17].

Furthermore, a systematic violation found in the structure calculations when there is sufficient NOE network anchoring may aid in identifying inappropriate angle restraints.

A general way to model structures using distance and angle restraints is by simulated annealing, as described above [86, 90]. The protocol of the calculation has been standardized. A completely unfolded state of target protein, namely at an extremely high temperature (e.g. 10,000 K), is virtually generated as an initial model, and the temperature is gradually cooled to around 0 K to minimize the total potential energy (or target functions) and to satisfying the input NMR restraints. It is well known that because the modeled NMR structures calculated by simulated annealing tend to be trapped into local minima of the energy landscape, the analysts should run multiple simulated annealing calculations with different random seeds to get an ensemble of modeled structures as mentioned below.

The structure calculation by simulated annealing using CYANA is performed using a highly simplified force field with smaller van der Waals radii and without static electric potential energy. In modern NMR structure determination, therefore, the models determined by CYANA are further refined by molecular dynamics calculations with explicit or implicit water model systems using other computer programs powered by advanced force fields, e.g., Xplor-NIH [123, 136], CNS [137], ARIA and related software [131, 132], AMBER [138], OPLS-AA [139] and CHARMM [140].

Conventionally, the final NMR structure is represented by an ensemble of 10–30 lowest-potential energy structures or structures with the lowest number of violations. Superposition of the structural ensemble can be performed manually based on the secondary structures in the determined structures. The automated identification of the ordered region of the determined structures is also possible using particular programs, e.g., NMRCORE [141], FindCore [142], CYRANGE [143] and FitRobot [144]. The resulting structural ensemble can be graphically represented using molecular viewers such as UCSF Chimera [145], CCP4mg [146], VMD-XPLOR [147], PyMol (https://www.pymol.org/) and MOLMOL [148]. The convergence of the modeled structures is estimated by the root-mean-square deviation (RMSD, Å) of backbone and/or heavy atoms of the 10–30 models that represent the ensemble [149, 150]. This RMSD provides an indicator of the precision of the models.

Regions where the structure is determined by a sufficient number of restraints appears well-converged, and therefore the RMSD value in that region is small. The local diversity of the RMSD values directly correlates to the number of restraints used in the structure calculation. Through superimposed representation of the structural ensemble it is easy to identify regions that poorly converge or are over-restrained. Poorly converged regions appear partially disordered or flexible. However, However, other possibilities may explain why the structure in that region could not be determined correctly in the modeled ensemble, i.e., insufficient restraints to force convergence of this region of the protein structure. Indicating whether the non-converged region is really disordered or dynamic in the deposition of the NMR structure into public databases or publication in a journal sometimes cannot be unambiguously stated. Thus, there is no conclusive evidence about whether the poorly converged region undergoes conformational fluctuations, without further investigation of the dynamics, kinetics and stoichiometrical features, which can be performed by NMR relaxation experiments.

**6. Validation of precision and accuracy of the determined protein structure**

Validating of the modeled structures to assess the precision and accurately is an important step in the structure determination process by NMR. There is no guarantee that the protein structure determined by solution NMR with high precision matches the same protein structure determined by a different method [149, 150]. To finalize the structural study by NMR, the analyst should carefully verify and assess both the precision and accuracy of the determined structure based on particular criteria.

As mentioned above, the convergence (precision) of the modeled structures can be generally assessed through the RMSD values of the 10–30 lowest minimal potential energy structures [151].

The accuracy of the modeled structures can be assessed through geometric parameters derived from the coordinates of the lowest energy structures [149, 150, 152]. The most widely used analysis examines bond angles, chirality and the side chain rotamer states, as well as the backbone conformation by creation of a Ramachandran plot. The results of the Ramachandran plot analysis are classified into four groups: 1) most favored, 2) additionally allowed, 3) generally allowed and 4) disallowed. Ideally the number of residues found in the generously allowed and disallowed regions of the Ramachandran plot is zero, or at least less than 10%, indicating that there are only a few geometric issues among the atoms and bonds in the structural ensemble [150]. This kind of analysis can be performed by PROCHECK-NMR [153, 154] or MolProbity [155], and is a requirement when depositing the molecular coordinates into the PDB.

The orientation of bond vectors in a protein, such as ^1^H^N^–^15^N or ^1^H_α_–^13^C_α_, relative to the principal axis of the molecular alignment tensor of the structure can be investigated by measuring residual dipolar coupling (RDC) between the dipolar-coupled nuclei using uniformly ^15^N- and/or ^13^C-labeled protein in a weakly aligned medium [152]. The information about the bond vector orientations in a protein can be used to validate the accuracy of the determined structure [152]. The interaction of the dipolar coupling between two spin-1/2 nuclei depends on the angle and distance between the bond vector and their gyromagnetic ratio. In contrast to solid-state NMR, the Hamiltonian derived from the dipolar coupling interaction between two dipolar-coupled nuclei is not observed because it averages to zero because of isotropic tumbling of the protein in solution. When the protein is dissolved together with a medium that orients under the influence of the magnetic field, e.g., a liquid crystalline formed by lipid discs such as bicelles [156], or filamentous phage such as Pf1 [157, 158], the aligned medium will provide some extent of anisotropy to the proteins owing to slight restriction of the isotropic molecular tumbling due to repetitive collisions between the protein and the aligned media [159]. As a result, a small residual value of the dipolar coupling between nuclei can be observed, and the size of the RDC can be tuned by the extent of alignment of the protein.

From the Cartesian coordinates of the aligned protein, it is theoretically possible to predict the alignment tensor of the weakly aligned protein using programs such as PALES [160] or REDCAT [161] by simulation of the predicted dipolar coupling between the bond vector and the principal axes of the estimated alignment tensor of the protein.

RDC values can also be calculated from the determined structure. The back-calculated RDC values from the structure (the RDC data have not been used as restraints in the structure calculation) should show a high correlation (correlation coefficient = 0.8–1.0) with the measured RDCs, confirming that the structure has been correctly determined [162]. Therefore, we would encourage this approach as a means to validate the protein structure, if the target protein is stable in alignment media and a sufficient number of RDCs are measured.

As aforementioned, if the analyst wants to directly apply the RDC values as restraints for structure calculation [159], it is necessary that the alignment tensor of the protein be determined precisely and unambiguously in advance. In a conventional manner, precise and unambiguous determination of the alignment tensor of the protein based on RDCs is impossible without information about the tertiary structure of the target protein. Therefore, conventionally, restraints for bond vector orientations using RDCs are generally used during the final refinement stage in NMR structure calculations [159]. Recently, however, methods for estimating the alignment tensor based solely on a histogram distribution of the experimental RDCs (without information about the tertiary structure) have been developed [163].

There is always a certain possibility that the determined structure with a high convergence is (locally or wholly) incorrect because internuclear distance restraints based on NOE and dihedral angle restraints fundamentally provide local (short-range, < 6 Å) information, which means those restraints cannot unambiguously restrict the relative orientation between each secondary structure or sub-domains of a protein [159]. In particular, in the cases of a multidomain protein or a protein-peptide complex, accurate determination of the relative orientation between each molecule is difficult when using such short-range restraints, even if the tertiary structure of each component has been determined precisely and accurately [164-167]. When the NMR structures have been refined using RDC data as restraints, as a matter of course, the RDC data may no longer be applied to validation.

**7. Concluding remark**

Solution NMR spectroscopy is a popular method to determine the tertiary structure of proteins. Surprisingly, however, there are only a handful of articles that describe the workflow of protein NMR structure determination [71, 168-170]. We anticipate that this mini-review should help scientists who are interested in protein solution structure determination by NMR.

The rapid and steady progress in NMR hardware and software, e.g., CYANA upgrade and automation of NMR signal assignment programs, has continued unabated [171]. Furthermore, recently, integrated NMR software platforms offering systematic and semi-automatic biomolecular structure determination, e.g. PONDEROSA-C/S [16, 18, 126] and UNIO [19, 87, 88, 95-97], have been developed. Moreover, global fold determination of desired proteins has now become easier owing to the development of programs, e.g. CS-ROSETTA [172, 173], which can determine protein structures using only chemical shifts and RDC data as restraints.

Furthermore, advanced techniques for protein sample preparation (e.g., SAIL technology [42, 174-177], methyl group-selective ^1^H,^13^C-labeling with deuteration of the other non-labile protons [178-183]) combined with elaborate NMR pulse schemes (e.g., rapid NMR data collection such as the Band Selective Optimized-Flip-Angle Short-Transient (SOFAST), Band-selective Excitation Short-Transient (BEST) [184, 185] and their easy set up/use scripts (http://www.ibs.fr/research/scientific-output/software/pulse-sequence-tools/), ASAP or ALSOFAST [186] methods, the 3D or 4D methyl-methyl NOESY based on high-resolution and diagonal-free HMQC-NOESY-HMQC pulse schemes [181, 187-189], and the dual- or parallel-FID acquisition approaches [190-192]), and non-uniform data sampling (NUS) applying NMR data collection of the indirect dimension and quantitative reconstitution of NMR spectra from sparsely sampled data [193-195], should facilitate the study of challenging proteins by NMR (e.g., membrane proteins, enzymes such as kinase/phosphatase, supramolecular complexes). Therefore, solution NMR spectroscopy is expected to develop even further as a tool for determining the tertiary structure of macromolecules and large molecular weight protein complexes (ca. > 50 kDa) [196-200].

In addition, solution NMR spectroscopy is a powerful tool for intermolecular interaction studies and screening of druggable seeds compounds with an atomic level resolution, because it can detect and determine a binding site, even if the interaction is extremely weak. By performing magnetization relaxation dispersion NMR experiments, tertiary structures of minor, excited or invisible protein conformation can be determined when this conformation is populated by <1% [201, 202]. Therefore, it is expected that methodological advances of solution NMR spectroscopy, which can determine the tertiary structure of a protein in the bound state accurately and precisely in a weak interaction, will become a unique and beneficial structural biology tool by maximizing the features of solution NMR spectroscopy. Experimentally determined internuclear distances and secondary structure information predicted by TALOS and its related programs [84, 85], CamShift [120] and SHIFTX2 [121] can greatly assist accurate homology based modeling or docking simulations to obtain structural models. The most successful examples using these methods would be HADDOCK [203, 204] and CS-ROSETTA [172, 173], whose feasibility has been generally accepted to yield structural insights that give rational explanations describing the biological functions of biomolecular complexes and models. The integration of the above methods and/or additional structural data such as X-ray scattering and information about intermolecular contacts derived from pull-down and yeast-two-hybrid assays as well as point mutation data, the so-called hybrid/integrative methods, is a new approach to study large biomacromolecular systems [205].

A number of software and web-based resources for NMR data analysis as described in this article are available and user friendly, and should help to open avenues for non-specialist and life scientists interested in studying the structure of proteins.

**Figure legend**

**Fig. 1** Workflow of protein structure determination by solution NMR spectroscopy. (A) First, target protein is uniformly labeled with NMR active isotopes (^13^C and ^15^N) and purified by chromatography processes. In many cases, it is essential to optimize the solution composition of the NMR sample (e.g., buffer, pH, type and concentration of salt, and other additives to prevent aggregation and/or to improve thermal stability of the target protein) and NMR parameters (e.g., sample temperature during NMR data collection, strength of static magnetic field, pulse sequences, inversion recovery delay) prior to collecting all the multidimensional heteronuclear NMR experiments. (B) Generally, various sample conditions and NMR parameters are verified by measuring 2D ^1^H-^15^N HSQC NMR spectra and/or 2D projection spectra of 3D HNCACB (e.g., 2D HN(CA)CB) until optimal conditions are found. If sample/NMR data collection optimization is performed by only ^15^N-based NMR experiments, only a ^15^N-labeled target protein (without ^13^C-labeling) is required. (C) The NMR signals of all ^1^H, ^13^C and ^15^N nuclei are assigned by analyzing multidimensional heteronuclear NMR spectra using NMR software that facilitates assignment of the signals. The right lower panel of Fig. 1 is a screenshot of a NMR signal assignment using the Kujira and MagRO software, developed by Prof. Naohiro Kobayashi (Osaka University, Japan), which is available at PDBj-BMRB <http://bmrbdep.pdbj.org/en/nmr_tool_box/magro_nmrview.html>). (D) After completing the assignment process, the solution structures of the target protein are determined by performing simulated annealing using NMR-based restraints such as the ^1^H–^1^H distances and dihedral angles derived from NOEs and chemical shifts, respectively. (E) Finally, the precision and the accuracy of the determined structure are verified statistically and experimentally (e.g., by analyzing the Ramachandran plot and measuring RDCs of the target protein, respectively).

**Acknowledgements**

We are grateful to Dr. Kyoko Furuita, Dr. Shoko Shinya, Prof. Young-Ho Lee (Osaka University, Japan), Dr. Yoshikazu Hattori (Tokushima Bunri University, Japan), and Prof. Chojiro Kojima (Yokohama National University, Japan) for many useful discussions and suggestions. This work was supported in part by the Platform Project for Supporting Drug Discovery and Life Science Research, and AMED-CREST from MEXT and AMED, Japan.

**References**

1. Ilari A and Savino C (2008) Protein structure determination by X-ray crystallography. Methods Mol Biol 452: 63-87.

2. Zhou ZH (2011) Atomic resolution cryo electron microscopy of macromolecular complexes. Adv Protein Chem Struct Biol 82: 1-35.

3. Wüthrich K (2001) The way to NMR structures of proteins. Nat Struct Biol 8: 923-925.

4. Marion D (2013) An introduction to biological NMR spectroscopy. Mol Cell Proteomics 12: 3006-3025.

5. Kempf JG and Loria JP (2003) Protein dynamics from solution NMR: theory and applications. Cell Biochem Biophys 37: 187-211.

6. Kovermann M, Rogne P, Wolf-Watz M (2016) Protein dynamics and function from solution state NMR spectroscopy. Q Rev Biophys 49: e6.

7. Jensen MR, Markwick PR, Meier S, Griesinger C, Zweckstetter M, Grzesiek S, Bernadó P, Blackledge M (2009) Quantitative determination of the conformational properties of partially folded and intrinsically disordered proteins using NMR dipolar couplings. Structure 17: 1169-1185.

8. Salmon L, Jensen MR, Bernadó P, Blackledge M (2012) Measurement and analysis of NMR residual dipolar couplings for the study of intrinsically disordered proteins. Methods Mol Biol 895: 115-125.

9. Gil S, Hošek T, Solyom Z, Kümmerle R, Brutscher B, Pierattelli R, Felli IC (2013) NMR spectroscopic studies of intrinsically disordered proteins at near-physiological conditions. Angew Chem Int Ed Engl 52: 11808-11812.

10. Jensen MR, Ruigrok RW, Blackledge M (2013) Describing intrinsically disordered proteins at atomic resolution by NMR. Curr Opin Struct Biol 23: 426-435.

11. Jensen MR, Zweckstetter M, Huang JR, Blackledge M (2014) Exploring free-energy landscapes of intrinsically disordered proteins at atomic resolution using NMR spectroscopy. Chem Rev 114: 6632-6660.

12. Abyzov A, Salvi N, Schneider R, Maurin D, Ruigrok RW, Jensen MR, Blackledge M (2016) Identification of dynamic modes in an intrinsically disordered protein using temperature-dependent NMR relaxation. J Am Chem Soc 138: 6240-6251.

13. Skinner AL and Laurence JS (2008) High-field solution NMR spectroscopy as a tool for assessing protein interactions with small molecule ligands. J Pharm Sci 97: 4670-4695.

14. Harner MJ, Frank AO, Fesik SW (2013) Fragment-based drug discovery using NMR spectroscopy. J Biomol NMR 56: 65-75.

15. Liu Z, Gong Z, Dong X, Tang C (2016) Transient protein-protein interactions visualized by solution NMR. Biochim Biophys Acta 1864: 115-122.

16. Braun W (1987) Distance geometry and related methods for protein structure determination from NMR data. Q Rev Biophys 19: 115-157.

17. Wüthrich K (1990) Protein structure determination in solution by NMR spectroscopy. J Biol Chem 265: 22059-22062.

18. Hiroaki H (2013) Recent applications of isotopic labeling for protein NMR in drug discovery. Expert Opin Drug Discov 8: 523-536.

19. Sugiki T, Fujiwara T, Kojima C (2014) Latest approaches for efficient protein production in drug discovery. Expert Opin Drug Discov 9: 1189-204.

20. Hiroaki H, Umetsu Y, Nabeshima Y, Hoshi M, Kohda D (2011) A simplified recipe for assigning amide NMR signals using combinatorial ^14^N amino acid inverse-labeling. J Struct Funct Genomics 12: 167-174.

21. Rasia R, Brutscher B, Plevin M (2012) Selective isotopic unlabeling of proteins using metabolic precursors: Application to NMR assignment of intrinsically disordered proteins. Chembiochem 13: 732-739.

22. Daly R and Hearn MT (2005) Expression of heterologous proteins in Pichia pastoris: a useful experimental tool in protein engineering and production. J Mol Recognit 18: 119-138.

23. Sugiki T, Shimada I, Takahashi H (2008) Stable isotope labeling of protein by Kluyveromyces lactis for NMR study. J Biomol NMR 42: 159-162.

24. Takahashi H and Shimada I (2010) Production of isotopically labeled heterologous proteins in non-E. coli prokaryotic and eukaryotic cells. J Biomol NMR 46: 3-10.

25. Sugiki T, Ichikawa O, Miyazawa-Onami M, Shimada I, Takahashi H (2012) Isotopic labeling of heterologous proteins in the yeast Pichia pastoris and Kluyveromyces lactis. Methods Mol Biol 831: 19-36.

26. Kigawa T, Yabuki T, Yoshida Y, Tsutsui M, Ito Y, Shibata T, Yokoyama S (1999) Cell-free production and stable-isotope labeling of milligram quantities of proteins. FEBS Lett 442: 15-19.

27. Ozawa K, Jergic S, Crowther J, Thompson P, Wijffels G, Otting G, Dixon N (2005) Cell-free protein synthesis in an autoinduction system for NMR studies of protein-protein interactions. J Biomol NMR 32: 235-241.

28. Matsuda T, Koshiba S, Tochio N, Seki E, Iwasaki N, Yabuki T, Inoue M, Yokoyama S, Kigawa T (2007) Improving cell-free protein synthesis for stable-isotope labeling. J Biomol NMR 37: 225-229.

29. Takai K, Sawasaki T, Endo Y (2010) Practical cell-free protein synthesis system using purified wheat embryos. Nat Protoc 5: 227-238.

30. Yokoyama J, Matsuda T, Koshiba S, Kigawa T (2010) An economical method for producing stable-isotope labeled proteins by the E. coli cell-free system. J Biomol NMR 48: 193-201.

31. Kainosho M, Torizawa T, Iwashita Y, Terauchi T, Mei Ono A, Güntert P (2006) Optimal isotope labelling for NMR protein structure determinations. Nature 440: 52-57.

32. Abe R, Shiraga K, Ebisu S, Takagi H, Hohsaka T (2010) Incorporation of fluorescent non-natural amino acids into N-terminal tag of proteins in cell-free translation and its dependence on position and neighboring codons. J Biosci Bioeng 110: 32-38.

33. Su XC, Loh CT, Qi R, Otting G (2011) Suppression of isotope scrambling in cell-free protein synthesis by broadband inhibition of PLP enymes for selective ^15^N-labelling and production of perdeuterated proteins in H_2_O. J Biomol NMR 50: 35-42.

34. Shimizu Y, Kuruma Y, Kanamori T, Ueda T (2014) The PURE system for protein production. Methods Mol Biol 1118: 275-284.

35. Deniaud A, Liguori L, Blesneac I, Lenormand JL, Pebay-Peyroula E (2010) Crystallization of the membrane protein hVDAC1 produced in cell-free system. Biochim Biophys Acta 1798: 1540-1546.

36. Reckel S, Sobhanifar S, Durst F, Löhr F, Shirokov VA, Dötsch V, Bernhard F (2010) Strategies for the cell-free expression of membrane proteins. Methods Mol Biol 607: 187-212.

37. Haberstock S, Roos C, Hoevels Y, Dötsch V, Schnapp G, Pautsch A, Bernhard F (2012) A systematic approach to increase the efficiency of membrane protein production in cell-free expression systems. Protein Expr Purif 82: 308-316.

38. Wang X, Wang J, Ge B (2013) Evaluation of cell-free expression system for the production of soluble and functional human GPCR N-formyl peptide receptors. Protein Pept Lett 20: 1272-1279.

39. Dutta A, Saxena K, Schwalbe H, Klein-Seetharaman J (2012) Isotope labeling in mammalian cells. Methods Mol Biol 831: 55-69.

40. Sastry M, Bewley CA, Kwong PD (2012) Mammalian expression of isotopically labeled proteins for NMR spectroscopy. Adv Exp Med Biol 992: 197-211.

41. Kikuchi J, Shinozaki K, Hirayama T (2004) Stable isotope labeling of Arabidopsis thaliana for an NMR-based metabolomics approach. Plant Cell Physiol 45: 1099-1104.

42. Chikayama E, Suto M, Nishihara T, Shinozaki K, Hirayama T, Kikuchi J (2008) Systematic NMR analysis of stable isotope labeled metabolite mixtures in plant and animal systems: coarse grained views of metabolic pathways. PLoS One 3: e3805.

43. Latham MP and Kay LE (2012) Is buffer a good proxy for a crowded cell-like environment? A comparative NMR study of calmodulin side-chain dynamics in buffer and E. coli lysate. PLoS One 7: e48226.

44. Tomita S, Ikeda S, Tsuda S, Someya N, Asano K, Kikuchi J, Chikayama E, Ono H, Sekiyama Y (2016) A survey of metabolic changes in potato leaves by NMR-based metabolic profiling in relation to resistance to late blight disease under field conditions. Magn Reson Chem. In press.

45. Zheng Z, Sun H, Hu C, Li G, Liu X, Chen P, Cui Y, Liu J, Wang J, Liang G (2016) Using "On/Off" (19)F NMR/magnetic resonance imaging signals to sense tyrosine kinase/phosphatase activity in vitro and in cell lysates. Anal Chem 88: 3363-3368.

46. Sugiki T, Yoshiura C, Kofuku Y, Ueda T, Shimada I, Takahashi H (2009) High-throughput screening of optimal solution conditions for structural biological studies by fluorescence correlation spectroscopy. Protein Sci 18: 1115-1120.

47. Horst R and Wüthrich K (2015) Micro-scale NMR Experiments for Monitoring the Optimization of Membrane Protein Solutions for Structural Biology. Bio Protoc 20: e1539.

48. Kozak S, Lercher L, Karanth MN, Meijers R, Carlomagno T, Boivin S (2016) Optimization of protein samples for NMR using thermal shift assays. J Biomol NMR 64: 281-289.

49. Flynn P, Mattiello D, Hill H, Wand A (2000) Optimal use of cryogenic probe technology in NMR studies of proteins. J Am Chem Soc 122: 4823-4824.

50. Rashid M, Gustafson K, Crouch R, Groweiss A, Pannell L, Van Q, Boyd M (2002) Application of high-field NMR and cryogenic probe technologies in the structural elucidation of poecillastrin A, a new antitumor macrolide lactam from the sponge Poecillastra species. Org Lett 4: 3293-3296.

51. Lee D, Hilty C, Wider G, Wüthrich K (2006) Effective rotational correlation times of proteins from NMR relaxation interference. J Magn Reson 178: 72-76.

52. Kay LE, Torchia DA, Bax A (1989) Backbone dynamics of proteins as studied by ^15^N inverse detected heteronuclear NMR spectroscopy: application to staphylococcal nuclease. Biochemistry 28: 8972-8979.

53. Yuwen T and Skrynnikov NR (2014) CP-HISQC: a better version of HSQC experiment for intrinsically disordered proteins under physiological conditions. J Biomol NMR 58: 175-192.

54. Driscoll PC, Clore GM, Marion D, Wingfield PT, Gronenborn AM (1990) Complete resonance assignment for the polypeptide backbone of interleukin 1 beta using three-dimensional heteronuclear NMR spectroscopy. Biochemistry 29: 3542-3556.

55. Clore GM, Bax A, Driscoll PC, Wingfield PT, Gronenborn AM (1990) Assignment of the side-chain ^1^H and ^13^C resonances of interleukin-1 beta using double- and triple-resonance heteronuclear three-dimensional NMR spectroscopy. Biochemistry 29: 8172-8184.

56. Kanelis V, Forman-Kay JD, Kay LE (2001) Multidimensional NMR methods for protein structure determination. IUBMB Life 52: 291-302.

57. Marion D, Driscoll PC, Kay LE, Wingfield PT, Bax A, Gronenborn AM, Clore GM (1989) Overcoming the overlap problem in the assignment of ^1^H NMR spectra of larger proteins by use of three-dimensional heteronuclear ^1^H-^15^N Hartmann-Hahn-multiple quantum coherence and nuclear Overhauser-multiple quantum coherence spectroscopy: application to interleukin 1 beta. Biochemistry 28: 6150-6156.

58. Fernández C and Wider G (2003) TROSY in NMR studies of the structure and function of large biological macromolecules. Curr Opin Struct Biol 13: 570-580.

59. Wider G (2005) NMR techniques used with very large biological macromolecules in solution. Methods Enzymol 394: 382-398.

60. Pervushin K, Riek R, Wider G, Wüthrich K (1997) Attenuated T2 relaxation by mutual cancellation of dipole-dipole coupling and chemical shift anisotropy indicates an avenue to NMR structures of very large biological macromolecules in solution. Proc Natl Acad Sci U S A 94: 12366-12371.

61. Bermel W, Bertini I, Duma L, Felli IC, Emsley L, Pierattelli R, Vasos PR (2005) Complete assignment of heteronuclear protein resonances by protonless NMR spectroscopy. Angew Chem Int Ed Engl 44: 3089-3092.

62. Bermel W, Bertini I, Felli IC, Lee YM, Luchinat C, Pierattelli R (2006) Protonless NMR experiments for sequence-specific assignment of backbone nuclei in unfolded proteins. J Am Chem Soc 128: 3918-3919.

63. Bermel W, Bertini I, Felli IC, Matzapetakis M, Pierattelli R, Theil EC, Turano P (2007) A method for C(alpha) direct-detection in protonless NMR. J Magn Reson 188: 301-310.

64. Bertini I, Jiménez B, Pierattelli R, Wedd AG, Xiao Z (2008) Protonless ^13^C direct detection NMR: characterization of the 37 kDa trimeric protein CutA1. Proteins 70: 1196-1205.

65. Wishart DS, Bigam CG, Yao J, Abildgaard F, Dyson HJ, Oldfield E, Markley JL, Sykes BD (1995) ^1^H, ^13^C and ^15^N chemical shift referencing in biomolecular NMR. J Biomol NMR 6: 135-140.

66. Markley JL, Bax A, Arata Y, Hilbers CW, Kaptein R, Sykes BD, Wright PE, Wüthrich K (1998) Recommendations for the presentation of NMR structures of proteins and nucleic acids. IUPAC-IUBMB-IUPAB Inter-Union Task Group on the Standardization of Data Bases of Protein and Nucleic Acid Structures Determined by NMR Spectroscopy. J Biomol NMR 12: 1-23.

67. Shen Y, Delaglio F, Cornilescu G, Bax A (2009) TALOS+: a hybrid method for predicting protein backbone torsion angles from NMR chemical shifts. J Biomol NMR 44: 213-223.

68. Shen Y and Bax A (2013) Protein backbone and sidechain torsion angles predicted from NMR chemical shifts using artificial neural networks. J Biomol NMR 56: 227-241.

69. Güntert P, Mumenthaler C, Wüthrich K (1997) Torsion angle dynamics for NMR structure calculation with the new program DYANA. J Mol Biol 273: 283-298.

70. Herrmann T, Güntert P, Wüthrich K (2002) Protein NMR structure determination with automated NOE assignment using the new software CANDID and the torsion angle dynamics algorithm DYANA. J Mol Biol 319: 209-227.

71. Güntert P (2004) Automated NMR structure calculation with CYANA. Methods Mol Biol 278: 353-378.

72. Johnson BA and Blevins RA (1994) NMR View: A computer program for the visualization and analysis of NMR data. J Biomol NMR 4: 603-614.

73. Kobayashi N, Iwahara J, Koshiba S, Tomizawa T, Tochio N, Güntert P, Kigawa T, Yokoyama S (2007) KUJIRA, a package of integrated modules for systematic and interactive analysis of NMR data directed to high-throughput NMR structure studies. J Biomol NMR 39: 31-52.

74. Kobayashi N, Harano Y, Tochio N, Nakatani E, Kigawa T, Yokoyama S, Mading S, Ulrich EL, Markley JL, Akutsu H, Fujiwara T (2012) An automated system designed for large scale NMR data deposition and annotation: application to over 600 assigned chemical shift data entries to the BioMagResBank from the Riken Structural Genomics/Proteomics Initiative internal database. J Biomol NMR 53: 311-320.

75. Jung YS and Zweckstetter M (2004) Mars -- robust automatic backbone assignment of proteins. J Biomol NMR 30: 11-23.

76. Rieping W, Habeck M, Bardiaux B, Bernard A, Malliavin TE, Nilges M (2007) ARIA2: automated NOE assignment and data integration in NMR structure calculation. Bioinformatics 23: 381-382.

77. Volk J, Herrmann T, Wüthrich K (2008) Automated sequence-specific protein NMR assignment using the memetic algorithm MATCH. J Biomol NMR 41: 127-138.

78. Fiorito F, Herrmann T, Damberger FF, Wüthrich K (2008) Automated amino acid side-chain NMR assignment of proteins using (13)C- and (15)N-resolved 3D [ (1)H, (1)H]-NOESY. J Biomol NMR 42: 23-33.

79. Herrmann T, Güntert P, Wüthrich K (2002) Protein NMR structure determination with automated NOE-identification in the NOESY spectra using the new software ATNOS. J Biomol NMR 24: 171-189.

80. Schmidt E and Güntert P (2012) A new algorithm for reliable and general NMR resonance assignment. J Am Chem Soc 134: 12817-12829.

81. Clore GM, Robien MA, Gronenborn AM (1993) Exploring the limits of precision and accuracy of protein structures determined by nuclear magnetic resonance spectroscopy. J Mol Biol 231: 82-102.

82. Furuita K, Kataoka S, Sugiki T, Hattori Y, Kobayashi N, Ikegami T, Shiozaki K, Fujiwara T, Kojima C (2015) Utilization of paramagnetic relaxation enhancements for high-resolution NMR structure determination of a soluble loop-rich protein with sparse NOE distance restraints. J Biomol NMR 61: 55-64.

83. Saio T, Ogura K, Yokochi M, Kobashigawa Y, Inagaki F (2009) Two-point anchoring of a lanthanide-binding peptide to a target protein enhances the paramagnetic anisotropic effect. J Biomol NMR 44: 157-166.

84. Saio T, Yokochi M, Kumeta H, Inagaki F (2010) PCS-based structure determination of protein-protein complexes. J Biomol NMR 46: 271-280.

85. Englander SW and Mayne L (1992) Protein folding studied using hydrogen-exchange labeling and two-dimensional NMR. Annu Rev Biophys Biomol Struct 21: 243-265.

86. Hwang TL, van Zijl PC, Mori S (1998) Accurate quantitation of water-amide proton exchange rates using the phase-modulated CLEAN chemical EXchange (CLEANEX-PM) approach with a Fast-HSQC (FHSQC) detection scheme. J Biomol NMR 11: 221-226.

87. Wang YX, Jacob J, Cordier F, Wingfield P, Stahl SJ, Lee-Huang S, Torchia D, Grzesiek S, Bax A (1999) Measurement of 3hJNC' connectivities across hydrogen bonds in a 30 kDa protein. J Biomol NMR 14: 181-184.

88. Cordier F and Grzesiek S (2002) Temperature-dependence of protein hydrogen bond properties as studied by high-resolution NMR. J Mol Biol 317: 739-752.

89. Karplus M (1963) Vicinal protein coupling in nuclear magnetic resonance. J Am Chem Soc 85: 2870-2871.

90. Vuister GW and Bax A (1992) Measurement of two-bond JCOH alpha coupling constants in proteins uniformly enriched with ^13^C. J Biomol NMR 2: 401-405.

91. Roche J, Ying J, Shen Y, Torchia DA, Bax A (2016) ARTSY-J: Convenient and precise measurement of (3)JHNHα couplings in medium-size proteins from TROSY-HSQC spectra. J Magn Reson 268: 73-81.

92. Archer S, Ikura M, Torchia D, Bax A (1991) An alternative 3D-NMR technique for correlating backbone N-15 with side-chain H-beta-resonances in larger proteins. J Magn Reson 95: 636-641.

93. Schwieters CD, Kuszewski JJ, Tjandra N, Clore GM (2003) The Xplor-NIH NMR molecular structure determination package. J Magn Reson 160: 65-73.

94. Schwieters C, Kuszewski J, Clore G (2006) Using Xplor-NIH for NMR molecular structure determination. Prog Nucl Magn Reson Spectrosc 48: 47-62.

95. Brunger A, Adams P, Clore G, DeLano W, Gros P, Grosse-Kunstleve R, Jiang J, Kuszewski J, Nilges M, Pannu N, Read R, Rice L, Simonson T, Warren G (1998) Crystallography & NMR system: A new software suite for macromolecular structure determination. Acta Crystallogr 54: 905-921.

96. Salomon-Ferrer R, Case D, Walker R (2013) An overview of the Amber biomolecular simulation package. Comp Mol Sci 3: 198-210.

97. Kobayashi N (2014) A robust method for quantitative identification of ordered cores in an ensemble of biomolecular structures by non-linear multi-dimensional scaling using inter-atomic distance variance matrix. J Biomol NMR 58: 61-67.

98. Koradi R, Billeter M, Wuthrich K (1996) MOLMOL: A program for display and analysis of macromolecular structures. J Mol Graph 14: 51-55.

99. Nabuurs S, Spronk C, Vriend G, Vuister G (2004) Concepts and tools for NMR restraint analysis and validation. Concept Magn Reson 22A: 90-105.

100. Spronk C, Nabuurs S, Krieger E, Vriend G, Vuister G (2004) Validation of protein structures derived by NMR spectroscopy. Prog Nucl Magn Reson Spectrosc 45: 315-337.

101. Huang X, Moy F, Powers R (2000) Evaluation of the utility of NMR structures determined from minimal NOE-based restraints for structure-based drug design, using MMP-1 as an example. Biochemistry 39: 13365-13375.

102. Rosato A, Tejero R, Montelione G (2013) Quality assessment of protein NMR structures. Curr Opin Struct Biol 23: 715-724.

103. Laskowski R, Macarthur M, Moss D, Thornton J (1993) PROCHECK - A program to check the stereochemical quality of protein structures. J Appl Crystallogr 26: 283-291.

104. Laskowski R, Rullmann J, MacArthur M, Kaptein R, Thornton J (1996) AQUA and PROCHECK-NMR: Programs for checking the quality of protein structures solved by NMR. J Biomol NMR 8: 477-486.

105. Bax A and Tjandra N (1997) High-resolution heteronuclear NMR of human ubiquitin in an aqueous liquid crystalline medium. J Biomol NMR 10: 289-292.

106. Hansen MR, Mueller L, Pardi A (1998) Tunable alignment of macromolecules by filamentous phage yields dipolar coupling interactions. Nat Struct Biol 5: 1065-1074.

107. Zweckstetter M and Bax A (2001) Characterization of molecular alignment in aqueous suspensions of Pf1 bacteriophage. J Biomol NMR 20: 365-377.

108. Lipsitz R and Tjandra N (2004) Residual dipolar couplings in NMR structure analysis. Annu Rev Biophys Biomol Struct 33: 387-413.

109. Zweckstetter M (2008) NMR: prediction of molecular alignment from structure using the PALES software. Nat Protoc 3: 679-690.

110. Schmidt C, Irausquin S, Valafar H (2013) Advances in the REDCAT software package. BMC Bioinform 14: 302.

111. Simon K, Xu J, Kim C, Skrynnikov N (2005) Estimating the accuracy of protein structures using residual dipolar couplings. J Biomol NMR 33: 83-93.

112. Fischer M, Losonczi J, Weaver J, Prestegard J (1999) Domain orientation and dynamics in multidomain proteins from residual dipolar couplings. Biochemistry 38: 9013-9022.

113. Bolon P, Al-Hashimi H, Prestegard J (1999) Residual dipolar coupling derived orientational constraints on ligand geometry in a 53 kDa protein-ligand complex. J Mol Biol 293: 107-115.

114. Jensen M, Ortega-Roldan J, Salmon L, van Nuland N, Blackledge M (2011) Characterizing weak protein-protein complexes by NMR residual dipolar couplings. Euro Biophys J Biophy Lett 40: 1371-1381.

115. Gobl C, Madl T, Simon B, Sattler M (2014) NMR approaches for structural analysis of multidomain proteins and complexes in solution. Prog Nucl Magn Reson Spectrosc 80: 26-63.

116. Guerry P and Herrmann T (2011) Advances in automated NMR protein structure determination. Q Rev Biophys 44: 257-309.

117. Takeda M, Ikeya T, Güntert P, Kainosho M (2007) Automated structure determination of proteins with the SAIL-FLYA NMR method. Nat Protoc 2: 2896-2902.

118. Ikeya T, Takeda M, Yoshida H, Terauchi T, Jee JG, Kainosho M, Güntert P (2009) Automated NMR structure determination of stereo-array isotope labeled ubiquitin from minimal sets of spectra using the SAIL-FLYA system. J Biomol NMR 44: 261-272.

119. Miyanoiri Y, Takeda M, Okuma K, Ono AM, Terauchi T, Kainosho M (2013) Differential isotope-labeling for Leu and Val residues in a protein by E. coli cellular expression using stereo-specifically methyl labeled amino acids. J Biomol NMR 57: 237-249.

120. Miyanoiri Y, Ishida Y, Takeda M, Terauchi T, Inouye M, Kainosho M (2016) Highly efficient residue-selective labeling with isotope-labeled Ile, Leu, and Val using a new auxotrophic E. coli strain. J Biomol NMR 65: 109-119.

121. Tugarinov V and Kay LE (2003) Ile, Leu, and Val methyl assignments of the 723-residue malate synthase G using a new labeling strategy and novel NMR methods. J Am Chem Soc 125: 13868-13878.

122. Tugarinov V, Kay L, Ibraghimov I, Orekhov V (2005) High-resolution four-dimensional H-1-C-13 NOE spectroscopy using methyl-TROSY, sparse data acquisition, and multidimensional decomposition. J Am Chem Soc 127: 2767-2775.

123. Hiller S, Ibraghimov I, Wagner G, Orekhov V (2009) Coupled decomposition of four-dimensional NOESY spectra. J Am Chem Soc 131: 12970-12978.

124. Loria J, Berlow R, Watt E (2008) Characterization of enzyme motions by solution NMR relaxation dispersion. Acc Chem Res 41: 214-221.

125. Anthis N and Clore G (2015) Visualizing transient dark states by NMR spectroscopy. Q Rev Biophys 48: 35-116.
